# Protein complex prediction based on *k*-connected subgraphs in protein interaction network

**DOI:** 10.1186/1752-0509-4-129

**Published:** 2010-09-16

**Authors:** Mahnaz Habibi, Changiz Eslahchi, Limsoon Wong

**Affiliations:** 1 Faculty of Mathematics, Shahid-Beheshti University, g.c., Tehran, Iran; 2 School of Computing, National University of Singapore, Singapore

## Abstract

**Background:**

Protein complexes play an important role in cellular mechanisms. Recently, several methods have been presented to predict protein complexes in a protein interaction network. In these methods, a protein complex is predicted as a dense subgraph of protein interactions. However, interactions data are incomplete and a protein complex does not have to be a complete or dense subgraph.

**Results:**

We propose a more appropriate protein complex prediction method, CFA, that is based on connectivity number on subgraphs. We evaluate CFA using several protein interaction networks on reference protein complexes in two benchmark data sets (MIPS and Aloy), containing 1142 and 61 known complexes respectively. We compare CFA to some existing protein complex prediction methods (CMC, MCL, PCP and RNSC) in terms of recall and precision. We show that CFA predicts more complexes correctly at a competitive level of precision.

**Conclusions:**

Many real complexes with different connectivity level in protein interaction network can be predicted based on connectivity number. Our CFA program and results are freely available from http://www.bioinf.cs.ipm.ir/softwares/cfa/CFA.rar.

## Background

Several groups have produced a large amount of data on protein interactions [1-9]. It is desirable to use this wealth of data to predict protein complexes. Several methods have been applied to protein inter-actome graphs to detect highly connected subgraphs and predict them as protein complexes [10-25]. The main criterion used for protein complex prediction is cliques or dense subgraphs. Spirin and Mirny proposed the clique-finding and super-paramagnetic clustering with Monte Carlo optimization approach to find clusters of proteins [10]. Another method is Molecular Complex Detection (MCODE) [11], which starts with vertex weighting and finds dense regions according to given parameters. On the other hand, the Markov CLuster algorithm (MCL) [26,27] simulates a flow on the network by using properties of the adjacency matrix. MCL partitions the graph by discriminating strong and weak flows in the graph. The next algorithm is RNSC (Restricted Neighborhood Search Clustering) [13]. It is a cost-based local search algorithm that explores the solution space to minimize a cost function, which is calculated based on the numbers of intra-cluster and inter-cluster edges.

However, many biological data sources contain noise and do not contain complete information due to limitations of experiments. Recently, some computational methods have estimated the reliability of individual interaction based on the topology of the protein interaction network (PPI network) [23,28,29]. The Protein Complex Prediction method (PCP) [30] uses indirect interactions and topological weight to augment protein-protein interactions, as well as to remove interactions with weights below a threshold. PCP employs clique finding on the modified PPI network, retaining the benefits of clique-based approaches. Liu et al. [31] proposed an iterative score method to assess the reliability of protein interactions and to predict new interactions. They then developed the Clustering based on Maximal Clique algorithm (CMC) that uses maximal cliques to discover complexes from weighted PPI networks.

Following these past works, we model the PPI network with a graph, where vertices represent proteins and edges represent interactions between proteins. We present a new algorithm CFA--short for *k*-Connected Finding Algorithm--to find protein complexes from this graph. Our algorithm is based on finding maximal k-connected subgraphs. The union of all maximal k-connected subgraphs (*k *≥ 1) forms the set of candidate protein clusters. These candidate clusters are then filtered to remove (i) clusters having less than four proteins and (ii) clusters having a large diameter. We compare the results of our algorithm with the results of MCL, RNSC, PCP and CMC. Our algorithm produces results that are comparable or better than these existing algorithms on real complexes of [32,33].

### Preliminaries

**Figure 1:**
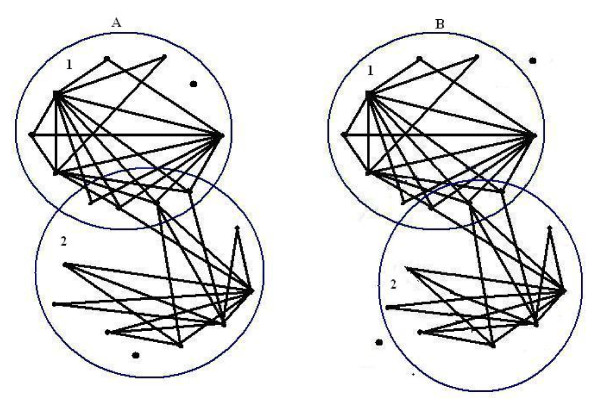
**Connectivity of two known complexes**. Part (A) contains two known complexes reported by MIPS (MIPS ID: 510.40.10 and 550.1.213). In complex 1, except for one vertex, there are at least two independent paths between every two proteins. In complex 2, except for two vertices, there are at least two independent paths between every two proteins. Part (B) are two 2-connected subgraphs obtained from the network in Part (A).

Generally, a complete or a dense subgraph of a protein interaction network is proposed to be a protein complex. But there are many complexes which have different topology and density (see Figure 1). So we need to define a criterion to predict protein complexes with different topology.

#### Interaction Graphs

A PPI network is considered as an undirected graph *G *= 〈*V*, *E*〉, where each vertex *v *∈ *V *represents a protein in the network and each edge *uv *∈ *E *represents an observed interaction between proteins *u *and *v*. Two vertices *u *and *v *of *G *are adjacent or neighbors if and only if *uv *is an edge of *G*. The degree *d*(*v*) of a vertex *v *is defined as the number of neighbors that the protein *v *has.

The density of a graph *G *= 〈*V*, *E*〉 is defined by

$$D_{G}=\frac{2|E|}{\left|V|(\;|V|-1\right)}$$

If all the vertices of *G *are pairwise adjacent, then *G *is a complete graph and *D_G _*= 1. A complete graph on *n *vertices is denoted by *K_n_*. The cluster score of *G *is defined as *D_G _*× |*V|*.

#### K-Connectivity

A path in a non-empty graph *G *= 〈*V*, *E*〉 between two vertices *u *and *v *is a sequence of distinct vertices *u *= *v_0_*, *v_1_*, ..., *v_k _*= *v *such that *v*_*i*_*v*_*i*+1 _∈ *E*, 0 ≤ *i < k *- 1. *G *is called connected if every two vertices of *G *are linked by a path in *G*. *G *is called *k*-connected (for *k *∈ ℵ) if |*V*| *> k *and the graph *G *= 〈*V *- *X*, *E *- (*X *× *X*)〉 is connected for every set *X *⊆ *V *with |*X*| *< k*. The distance *d*(*u*, *v*) is the shortest path in *G *between two vertices *u *and *v*. The greatest distance between any two vertices in *G *is the diameter of *G *denoted by *diamG*. A non-empty 1-connected subgraph with the minimum number of edges is called a tree. It is well known that a connected graph is a tree if and only if the number of edges of the graph is one less than the number of its vertices. It is a classic result of graph theory-- the global version of Menger's theorem [34]--that a graph is *k*-connected if any two of its vertices can be joined by *k *independent paths (two paths are independent if they only intersect in their ends).

## Results and Discussion

### Data Sets

#### Protein-Protein Interaction Network Data

In this work, we use two high-throughput protein-protein interaction (PPI) data collections. The first data collection, GRID, contains six protein interaction networks from the *Saccharomyces cerevisiae *(bakers' yeast) genome. These include two-hybrid interactions from Uetz et al. [2] and Ito et al. [3], as well as interactions characterized by mass spectrometry technique from Ho, Gavin, Krogan and their colleagues [6-9]. We refer to these data sets as *PPI_Uetz_*, *PPI_Ito_*, *PPI_Ho_*, *PPI_Gavin2_*, *PPI_Gavin6_*, and *PPI_Krogan_*.

The other data collection is obtained from BioGRID [35]. This data collection includes interactions obtained by several techniques. We only consider interactions derived from mass spectrometry and two-hybrid experiments as these represent physical interactions and co-complexed proteins. We refer to this data set as *PPI_BioGRID_*. Some descriptive statistics of each protein interaction network are presented in Table 1.

**Table 1:** Summary statistics of each data set.

Data set	Proteins	Interactions	Min. Deg	Avg.Deg	Max. Deg
*PPI_BioGRID_*	5040	27557	0	10.93	318
*PPI*_*Gavin*6_	1563	6531	0	8.36	81
*PPI*_*Gavin*2_	1373	3200	0	4.66	52
*PPI_Krogan_*	2672	7073	0	5.29	140
*PPI_Ho_*	1563	3596	1	4.60	62
*PPI_Ito_*	775	732	0	1.8	54
*PPI_Uetz_*	823	823	0	1.7	21

#### Protein Complex Data

Two reference sets of protein complexes are used in our work. The first data set was gathered by Aloy et al. [32] and the other was released in the Munich Information Center for Protein Sequences (MIPS) [33] at the time of this work (September 2009). We refer to the two protein complex data sets as APC (Aloy Protein Complex) and MPC (MIPS Protein Complex), respectively. Details of these data sets are described in Table 2. During validation, those proteins which cannot be found in the input interaction network are removed from the complex data.

**Table 2:** Summary statistics of each protein complex data sets for each PPI network.

PPI	MPC			APC		
		
	No. of Complex	Avg. Size	Max size	No. of Complex	Avg. Size	Max size
*Biogrid*	651	11.94	88	62	9.29	34
*Gavin*6	443	11.31	80	53	8.84	27
*Gavin*2	439	11.35	88	54	8.72	26
*Krogan*	531	10.89	75	56	8.94	31
*Ho*	543	10.55	70	30	6.60	18
*Ito*	119	5.85	20	15	4.86	8
*Uetz*	355	9.15	56	12	6.41	14

#### Cellular Component Annotation

The level of noise in protein interaction data--especially those obtained by two-hybrid experiments--has been estimated to be as high as 50% [36-38]. Liu et al. [31] have shown that using a de-noised protein interaction network as input leads to better quality of protein complex predictions by existing methods. A protein complex can only be formed if its proteins are localized within the same component of the cell. So we use localization coherence of proteins to clean up the input protein interaction network. We use cellular component terms from Gene Ontology (GO) [39] to evaluate localization coherence. We find that among the 5040 yeast proteins, only 4345 or 86% of them are annotated. To avoid arriving at misleading conclusions caused by biases in the annotations, we use the concept of informative cellular component. We define a cellular component annotation as informative if it has at least k proteins annotated with it and each of its descendent GO terms has less than k proteins annotated with it. In this work, we set k as 10. This yields 150 informative cellular component GO terms on the BioGRID data set.

### Performance Evaluation Measures

There are many studies that predict protein complexes. To evaluate the performance of various protein complex prediction methods, we compare the predicted protein complexes with real protein complex data sets, APC and MPC.

To compare the clusters--i.e., predicted protein complexes--found by different algorithms to real protein complexes, we use a measure based on the fraction of proteins in the predicted cluster that overlaps with the known complex. Let *S *be a predicted cluster and *C *be a reference complex, with size |*S*| and |*C*| respectively. The matching score between *S *and *C *is defined by

$$Overlap(S,C)=\frac{|S\cap C{|}^{2}}{\left|S||C\right|}$$

If *Overlap*(*S*,*C*) meets or exceeds a threshold *θ*, then we say *S *and *C *match. Following Liu et al. [31], we use an overlap threshold of 0.5 to determine a match.

Given a set of reference complexes *C *= {*C*_1_, *C*_2_, ...., *C_n_*}and a set of predicted complexes *S *= {*S*_1_,*S*_2_, ..., *S_m_*}, precision and recall at the whole-complex level are defined as follows:

$$\begin{array}{l} Prec=\frac{\left|\{S_{i}\in S|\exists C_{j}\in C,\;Overlap(S_{i},C_{j})\geq \theta \}\right|}{\left|S\right|}\\ Recall\;=\frac{\left|\{C_{i}\in C|\exists S_{j}\in S,\;Overlap(S_{j},C_{i})\geq \theta \}\right|}{\left|C\right|} \end{array}$$

The precision and recall are two numbers between 0 and 1. They are the commonly used measures to evaluate the performance of protein complex prediction methods [30,31]. In particular, precision corresponds to the fraction of predicted clusters that matches real protein complexes; and recall corresponds to the fraction of real protein complexes that are matched by predicted clusters.

Another measure which can be used to evaluate the performance of a method is *F*-measure. According to [40], this measure was first introduced by Rijsbergen [41]. They defined *F*-measure as the harmonic mean of precision and recall:

$$F=\frac{2*Prec*Recall}{Prec+Recall}$$

### Observations

To justify using the connectivity definition and cellular component annotation, we analyze the connectivity number and localization coherence of reference complexes of MPC on PPI networks obtained by [6-9] as well as [35].

#### Co-Localization Score of Known Complexes

A protein complex is a set of proteins that interact with each other at the same time and place, forming a single multimolecular machine [10]. This biological definition of a protein complex helps us predict protein complexes. Using the information of cellular component annotation existing in GO, Liu et al. [31] define a localization group as the set of proteins annotated with a common informative cellular component GO annotation. They then define the co-localization score of the complex, *c*, as the maximum number of proteins in the complex that are in the same localization group, *max*{*c *∩ *L_i _*| *i *= 1, ...,*k*}, divided by the number of those proteins in *c *with localization annotations, |{*p *∈ *c*|∃*L_i _*∈ *L*, *p *∈ *L_i_*}|, where *L *= {*L*_1_, ..., *L_k _*}is a set of localization groups. More formally, the co-localization score of a set of complexes *C *is the weighted average score over all complexes:

$$locscore(C)=\frac{\sum_{c\in C}max\{c\cap L_{i}|i=1,\ldots ,k\}}{\sum_{c\in C}|\{p\in c|\exists L_{i}\in L,p\in L_{i}\}|}$$

The *locscore *for MPC and APC are 0.74 and 0.86 respectively. The relatively large values of these numbers suggest that cleaning the input PPI network by cellular component information should help us improve precision and recall of existing algorithms.

#### Impact of Localization Information

In this work, the cleaning of PPI networks using informative cellular component GO terms is an important preprocessing step. So we analyze here the impact of using informative GO cellular component annotation on the performance of four existing algorithms--CMC, MCL, PCP, and RNSC-- on their standard parameters. (The CMC package comes with its own PPI-cleaning method. However, in order to observe the effect of cleaning based on cellular component GO terms on CMC, this method is not used in this work.)

Let *G_i _*= *G*[*L_i_*] be the induced subgraph of *G *generated by the vertex set *L_i_*, where {*L*_1_, *L*_2_, ..., *L_k_*} is the set of localization groups. Thus each *L_i _*contains a set of proteins localized to the same cellular component--i.e., they are annotated by the same informative GO term. Let *C_i _*be the set of all clusters predicted by an algorithm on $G_{i}\cdot \;C_{L}=\cup_{i=1}^{k}C_{i}$ denotes the set of all clusters predicted by the algorithm on *G*.

To evaluate the impact of localization information, we compare the precision and recall of *C_L _*and clusters generated on the original PPI network *G*. Table 3 summarizes some general features of clusters predicted by the algorithms mentioned. We observe that, by using protein cellular component annotations, the number of predicted clusters generally increases, while the average cluster size decreases. We further observe that the average size of clusters predicted by MCL and CMC algorithms are larger than those predicted by others. We also compare the precision and recall of the clusters predicted by the four algorithms. We find that generally the precision and recall values have significant improvements in *C_L_*.

**Table 3:** Features of clusters predicted by different algorithms on the both the original and *C*_*L *_networks.

		CMC					MCL				
			
			Avg			Avg		Avg			Avg
*PPI*	Setting	Cluster	Size	Prec	Recall	**Den**.	Cluster	Size	Prec	Recall	**Den**.
*BioGRID*	(1)	295	9.58	0.210	0.124	0.78	376	10.41	0.098	0.072	0.40
	(2)	296	9.36	**0.361**	**0.155**	0.75	647	9.43	**0.2411**	**0.173**	0.49
*Gavin*6	(1)	155	10.51	0.367	0.203	0.66	160	11.72	0.471	0.194	0.50
	(2)	299	9.55	**0.401**	**0.239**	0.62	327	9.40	**0.486**	**0.261**	0.57
*Gavin*2	(1)	110	11.5	0.390	0.118	0.44	115	9.81	**0.652**	0.252	0.39
	(2)	213	9.69	**0.417**	**0.159**	0.49	373	9.63	0.479	**0.266**	0.40
*Krogan*	(1)	215	8.93	0.251	0.124	0.60	246	8.07	0.146	0.094	0.43
	(2)	166	8.15	**0.494**	**0.163**	0.64	247	7.77	**0.477**	**0.184**	0.54
*Ho*	(1)	121	8.17	0.206	0.057	0.32	146	8.19	0.486	**0.145**	0.34
	(2)	149	7.89	**0.335**	**0.103**	0.45	96	7.28	**0.500**	0.110	0.36

		**PCP**					**RNSC**				
			
			**Avg**			**Avg**		**Avg**			**Avg**
***PPI***	**Setting**	**Cluster**	**Size**	**Prec**	**Recall**	**Den**.	**Cluster**	**Size**	**Prec**	**Recall**	**Den**.

*BioGRID*	(1)	174	8.73	0.253	0.109	0.51	174	6.31	0.367	0.119	0.78
	(2)	341	9.27	**0.343**	**0.158**	0.60	301	7.36	**0.425**	**0.156**	0.81
*Gavin*6	(1)	95	9.61	0.463	0.185	0.63	105	6.41	0.381	0.126	0.74
	(2)	228	9.14	**0.482**	**0.243**	0.62	295	7.33	**0.410**	**0.234**	0.72
*Gavin*2	(1)	54	9.40	**0.537**	0.125	0.50	89	5.98	0.370	0.074	0.61
	(2)	121	9.17	0.446	**0.141**	0.45	158	6.81	**0.487**	**0.132**	0.59
*Krogan*	(1)	100	7.90	0.380	0.109	0.61	92	6.25	0.423	0.081	0.72
	(2)	205	7.66	**0.458**	**0.158**	0.68	200	6.77	**0.510**	**0.165**	0.69
*Ho*	(1)	42	5.59	0.285	0.040	0.29	15	6.85	0.333	0.046	0.41
	(2)	51	5.00	**0.372**	**0.060**	0.37	26	6.84	**0.370**	**0.073**	0.38

In the setting column, (1) refers to the original network and (2) refers the network obtained by seggregation according to informative cellular component GO term annotations.

The precision and recall values obtained at the matching threshold *θ *= 0.5 are given in Table 3. RNSC performs best on *PPI_Biogrid_*, while MCL performs best on *PPI*_*Gavin*6_, *PPI*_*Gavin*2_, and *PPI_Ho_*. In the orginal network of *PPI_krogan_*, PCP shows better precision against recall compared to other methods, while after cleaning by using localization information almost all methods have similar performance. This table shows that none of these algorithms has the best precision vs recall in all networks.

**Figure 2:**
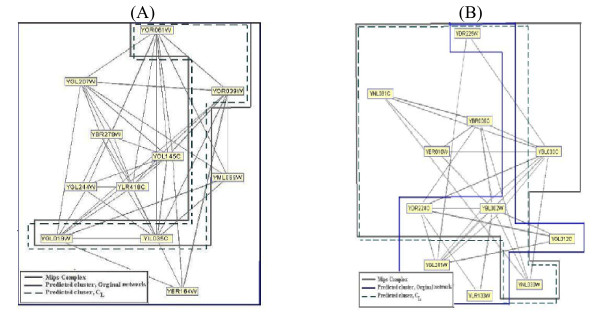
**Examples of the clusters predicted on the original and the *C_L_*. networks**. Part (A) illustrates the impact of using informative cellular component GO term annotations on the performance of CMC. CMC predicts the unmatched cluster on the original network. This cluster is refined in *C_L _*to matched well with the real complex in MPC. Part (B) shows a seven-member cluster predicted by PCP after the input PPI network is cleansed using informative cellular component GO term annotations.

We present two illustrative examples in Figure 2. The first example (Figure 2(A)) is the unmatched cluster predicted by CMC on the original network of *PPI*_*Gavin*2_. This cluster contains a four-member protein complex with specific GO cellular component annotation (GO.0005956; protein kinase CK2 complex). The other seven proteins in the CMC cluster belong to other localization groups. This cluster is refined in *C_L _*to match well with the same real complex. In Figure 2(B), PCP predicts a sevenmember cluster matched to a complex of MPC using the localization annotation on *PPI_Krogan_*. In contrast, only four proteins in this complex are matched to the corresponding PCP cluster predicted on the original network.

#### Density of Known Complexes

**Figure 3:**
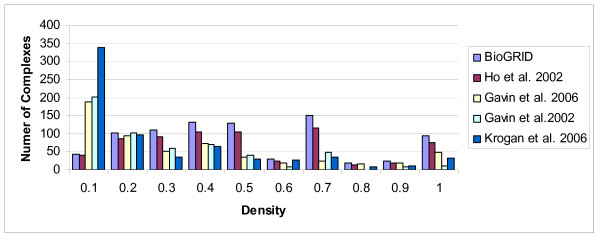
**The frequency distribution of known protein complexes having various density of protein interactions within them**.

We consider the density of known complexes with size at least three for each PPI network. Figure 3 shows that algorithms based on graph density cannot predict a large number of known complexes, and recall values of these algorithms are destined to be limited. For example, there are 11 complexes among 827 known complexes with *D_G _*= 0 and 41 complexes with density value less than 0.1 in *PPI_BioGRID_*. Similarly, there exist 200 complexes among 551 known complexes with density value less than 0.1 in *PPI*_*Gavin*2_.

Furthermore, almost all complexes which are complete or have high density are of the form *K*_3_, while there are a large number of cliques of size 3 which are not complex. For example, in *PPI_BioGRID_*, there exist 176 known complexes of size three, while the number of cliques of size 3 in *PPI_BioGRID _*is 37230. It means that only about 0.47% of them are known real complexes. So, those clusters and complexes with size atmost three are removed in our work, to avoid an excessive number of false positive predictions.

We have also studied the number of known complexes of size four in *PPI_BioGRID_*. We find that there exist 138 real complexes of size four, while only 54 of them have high density.

The discussions above suggest that the density criterion alone cannot answer the question of finding complexes. We need to introduce another criterion to overcome this problem.

#### Connectivity of Known Complexes

We show in this section that connectivity is a reasonable alternative criterion for identifying protein complexes. Although this criterion is simple, it may directly describe the general understanding of the protein complex concept. This criterion is better than density because, while there are a lot of known complexes that are not complete or dense, there are many k-connected subgraphs with low density. For example, Figure 1(A) shows two real complexes of MPC with low density (0.34). Both of them have a large 2-connected subgraph.

Similar to the definition of *locscore*, we define *kscore *of a set of complexes, *C*, as follows;

$$kscore(C)=\frac{\sum_{c\in C}max\{|s_{i}^{k}(c)|\;\;|i=1,\ldots ,n\}}{\sum_{c\in C}|\{p\in c|\exists q\in c,pq\in E\}|}$$

where $s_{1}^{k}(c),\;s_{2}^{k}(c),\;\;...,\;\;s_{n}^{k}(c)$ are maximal *k*- connected subgraphs of complex *c*.

In Table 4, the *kscore *and average density of different PPI networks on MPC are shown. The average density of the set of real complexes are usually low. On the other hand, on average, 99.5% of proteins of each real complex are located in 1-connected subgraphs. Also 78.4%, 53.7% and 37.4% of proteins of each real complex are located in 2-connected, 3- connected, and 4-connected subgraphs respectively. By increasing the connectivity number, this average decreases but there exist some proteins which are located in a subset of a real complex with high *k*- connectivity.

**Table 4:** The *kscore *and average density of different PPI networks on MPC.

Data Set	*PPI_BioGRID_*	*PPI*_*Gavin*6_	*PPI*_*Gavin*2_	*PPI_Krogan_*	*PPI_Ho_*
Avg Density	0.41	0.29	0.21	0.20	0.25
1*score*	0.995	0.929	0.970	0.870	0.983
2*score*	0.784	0.868	0.758	0.678	0.748
3*score*	0.537	0.521	0.494	0.351	0.446
4*score*	0.374	0.318	0.397	0.254	0.232

This suggests that using connectivity number as a criterion of protein complex prediction may be a good approach. Therefore, our algorithm is based on finding maximal k-connected subgraphs in PPI networks by keep increasing *k *until *k *cannot be increased any more. In other words, the algorithm continues until some integer *k*_0 _such that there is no k-connected subgraph with *k > k*_0_.

### Evaluation

#### Testing for Accuracy

To check the validity of CFA, we compare clusters predicted by CFA with the clusters obtained by CMC, MCL, PCP and RNSC, on the seven protein interaction networks of GRID and BioGRID. The networks are first segregated by informative cellular component GO terms before these algorithms are run. MPC and APC are used as benchmark real protein complexes.

In *PPI_Uetz_*, none of the algorithms could produce any cluster matched by real complexes in MPC and APC. *PPI_U etz _*is a difficult example because, as can be seen in Table 1, it is a much sparser and much more incomplete network compared to the other PPI networks. So in Table 5, we present the number of matched clusters and matched complexes predicted by the clustering methods on the other six PPI networks.

**Table 5:** Precision and recall values of different algorithms on each PPI network.

			APC			MPC		
				
Method	Data Set	No. of Cluster	Match Complex	Recall/Prec	Match Cluster	Match Complex	Recall/Prec	Match Cluster
CFA	(1)	423	52	**0.838 **0.310	131	119	**0.182 0.435**	184
CMC	(1)	296	51	0.822 0.293	87	101	0.155 0.361	107
MCL	(1)	647	51	0.822 0.179	116	113	0.173 0.241	156
PCP	(1)	341	50	0.806 0.290	99	103	0.158 0.343	117
RNSC	(1)	301	52	0.838 **0.345**	104	102	0.156 0.425	128

CFA	(2)	324	51	**0.962 0.456**	148	122	**0.275 0.543**	176
CMC	(2)	299	50	0.943 0.347	104	106	0.239 0.401	120
MCL	(2)	327	50	0.943 0.422	138	116	0.261 0.486	159
PCP	(2)	228	48	0.905 0.403	92	108	0.243 0.482	110
RNSC	(2)	295	50	0.943 0.362	107	104	0.234 0.410	121

CFA	(3)	235	49	**0.907 0.497**	117	119	**0.271 0.595**	140
CMC	(3)	213	31	0.574 0.347	74	70	0.159 0.417	89
MCL	(3)	373	47	0.870 0.332	124	117	0.266 0.479	179
PCP	(3)	121	28	0.518 0.388	47	62	0.141 0.446	54
RNSC	(3)	158	25	0.463 0.392	62	58	0.132 0.487	77

CFA	(4)	330	45	**0.803 0.451**	149	104	**0.195 0.533**	176
CMC	(4)	166	40	0.714 0.379	63	87	0.163 0.494	82
MCL	(4)	247	45	0.803 0.368	91	98	0.184 0.477	118
PCP	(4)	205	40	0.714 0.400	82	84	0.158 0.458	94
RNSC	(4)	200	37	0.660 0.430	86	88	0.165 0.510	102

CFA	(5)	120	13	**0.433 **0.166	20	62	**0.114 **0.416	50
CMC	(5)	149	6	0.200 0.060	9	56	0.103 0.335	50
MCL	(5)	96	12	0.400 0.250	24	60	0.110 0.500	48
PCP	(5)	51	4	0.133 0.098	5	33	0.060 0.372	19
RNSC	(5)	26	5	0.166 **0.500**	13	21	0.038 **0.730**	19

CFA	(6)	45	3	**0.200 0.088**	4	15	**0.126 **0.226	12
CMC	(6)	9	0	0.000 0.000	0	1	0.008 0.111	1
MCL	(6)	65	3	0.200 0.076	5	15	0.126 0.230	15
PCP	(6)	8	0	0.000 0.000	0	1	0.008 0.125	1
RNSC	(6)	11	0	0.000 0.000	0	1	0.008 **0.545**	6

The "data sets" column refers to networks, where (1) denotes *PPI_Biogrid_*, (2) denotes *PPI*_*Gavin*6_, (3) denotes *PPI*_*Gavin*2_, (4) denotes *PPI_Krogan_*, (5) denotes *PPI_Ho_*, and (6) denotes *PPI_Ito_*. The best precision and recall value for each PPI network are highlighted in bold font.

Table 5 shows that CFA performs better on *PPI_Krogan_*, *PPI_Ito_*, *PPI*_*Gavin*2 _and *PPI*_*Gavin*6 _compared to other methods. In fact, both precision and recall values of CFA are greater than all of the other algorithms in these networks. In *PPI_Ho_*, RNSC has the greatest precision. However, RNSC predicts merely 26 clusters and, among these predictions, 13 clusters are matched to 5 real complexes in APC and 19 clusters are matched to 21 real complexes in MPC. Thus the recall value of RNSC is very low (0.166 on APC and 0.038 on MPC). In contrast, CFA correctly predicts 13 real complexes of APC and 62 of MPC. The clusters of CFA give the precision value 0.416 (0.166) and the recall value 0.114 (0.433) on MPC (APC), which are generally better than that obtained by RNSC and other methods on *PPI_Ho_*.

We also study the number of matched clusters and matched complexes of predictions on *PPI_Biogrid_*. We find that almost all algorithms predict the same number of real complexes in APC. However, CFA matches a lot more complexes in MPC than CMC (18% more), MCL (5% more), PCP (15% more) and RNSC (17% more). Furthermore, this significant superiority of CFA in recall comes with the highest precision value in MPC. The overall precision of CFA on the combined APC and MPC complexes, as can be computed from Table 6, is 0.492, which is comparable to CMC (0.422), PCP (0.411), and RNSC (0.502), and is superior to MCL (0.274).

**Table 6:** Detailed breakdown of predicted clusters by different algorithms with respect to APC and MPC reference protein complexes.

Method	|*A*|	|*B*|	|*A *∪ *B*|	|*A *- *B*|	|*B *- *A*|	No. of Cluster	Precision
CFA	184	131	208	**77**	**24**	423	0.492
CMC	107	87	125	38	18	296	0.422
MCL	156	116	177	61	21	647	0.274
PCP	117	99	140	41	23	341	0.411
RNSC	128	104	151	47	23	301	0.502

Here A is the set of matched clusters on MPC and B is the set of matched clusters on APC. The number of matched clusters on MPC or APC, exclusively MPC and exclusively APC are shown.

**Figure 4:**
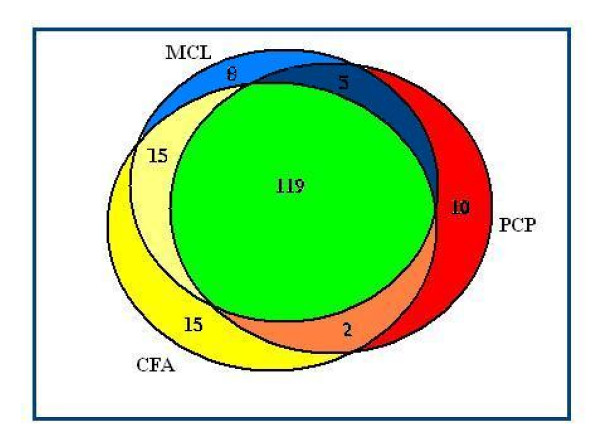
**The Venn diagram of matched complexes**. A Venn diagram of the combined set of complexes in APC and MPC that are correctly predicted by CFA, CMC and RNSC based on *PPI_Biogrid _*network.

We find that all complexes predicted by CMC and RNSC are identified by at least one of the other three algorithms. To compare real complexes predicted by CFA, MCL and PCP, Figure 4 shows a Venn diagram of complexes predicted by these algorithms on the combined set of APC and MPC complexes. It shows that CFA predicts maximum number of real complexes that MCL and PCP cannot predict. So CFA is finding a different group of complexes from other methods.

Some interactions in *PPI_Biogrid _*are derived from two-hybrid technique. Due to the level of noise in two-hybrid experiments, we expect those predicted clusters having the form of a tree structure to have lower reliability compared to other 1-connected subgraphs. Hence, in order to improve the results of CFA, we only use 1-connected subgraphs that are not trees. A tree with *n *vertices has *n *- 1 edges; so a connected cluster is a tree if and only if its cluster score is 2. Thus, we consider 1-connected subgraphs with cluster scores greater than 2. Similarly, we can do additional filtering for each k-connected subgraphs by considering the clusters with cluster score greater that *k*+1. The precision and recall values of the resulting further refined clusters are 0.465 and 0.178 in MPC and 0.347 and 0.838 in APC. So the precision vs recall of CFA, using cluster score filtering, shows significant improvement compared to other methods in *PPI_Biogrid _*on APC too.

On the other hand, we observe that some predicted clusters have large overlap with each other. That is, we have some clusters *S_i _*and *S_j _*such that *Overlap*(*S*_*i*, _*S_j_*) ≥ *α*. To get a more concise understanding of CFA and the other prediction methods, we also clean up the set of predictions by removing redundant clusters. In the other words, when two predicted clusters show an overlap score above the threshold value (of *α *= 0.5), we keep the larger one. The precision and recall values after this additional cleaning of the set of predictions are given in Table 7. Table 7 shows that, generally, CFA identifies the most number of complexes based on nonredundant predicted clusters on each PPI network.

**Table 7:** Precision and recall values after removing highly overlapping clusters.

			*APC*			*MPC*		
				
Method	Data Set	No. of Cluster	Recall/Prec/F-measure			Recall/Prec/F-measure		
CFA	(1)	238	**0.822**	**0.277**	**0.415**	**0.170**	**0.378**	**0.235**
CMC	(1)	208	0.741	0.235	0.358	0.145	0.322	0.200
MCL	(1)	467	0.790	0.113	0.199	0.147	0.164	0.155
PCP	(1)	230	0.758	0.226	0.348	0.133	0.282	0.181
RNSC	(1)	186	0.809	0.274	0.409	0.150	0.365	0.213

CFA	(2)	164	**0.924**	**0.390**	**0.549**	**0.250**	**0.530**	**0.340**
CMC	(2)	197	**0.924**	0.274	0.423	0.214	0.355	0.267
MCL	(2)	191	0.905	0.272	0.419	0.221	0.356	0.272
PCP	(2)	144	0.811	0.319	0.458	0.214	0.416	0.283
RNSC	(2)	152	**0.924**	0.348	0.506	0.205	0.263	0.230

CFA	(3)	124	**0.907**	0.475	**0.624**	**0.250**	**0.564**	**0.347**
CMC	(3)	122	0.500	0.237	0.322	0.123	0.295	0.173
MCL	(3)	215	0.851	0.237	0.371	0.248	0.395	0.305
PCP	(3)	82	0.481	**0.487**	0.485	0.116	0.365	0.176
RNSC	(3)	90	0.425	0.255	0.320	0.120	0.377	0.183

CFA	(4)	169	**0.767**	0.337	0.469	**0.180**	**0.455**	**0.258**
CMC	(4)	120	0.714	0.341	0.462	0.158	0.450	0.234
MCL	(4)	150	**0.767**	0.293	0.425	0.169	0.386	0.235
PCP	(4)	130	0.678	0.300	0.416	0.133	0.369	0.196
RNSC	(4)	108	0.660	**0.370**	**0.475**	0.160	0.500	0.242

CFA	(5)	96	**0.400**	0.156	0.225	**0.105**	0.385	0.165
CMC	(5)	109	0.166	0.045	0.072	0.073	0.247	0.113
MCL	(5)	71	**0.400**	0.169	**0.238**	**0.105**	**0.408**	**0.167**
PCP	(5)	43	0.133	0.093	0.110	0.055	0.325	0.094
RNSC	(5)	16	0.166	**0.312**	0.217	0.023	0.562	0.045

CFA	(6)	41	**0.134**	0.049	0.071	**0.126**	0.195	**0.153**
CMC	(6)	8	0.000	0.000	0.000	0.008	0.125	0.015
MCL	(6)	52	**0.134**	**0.076**	**0.097**	0.117	0.192	0.145
PCP	(6)	8	0.000	0.000	0.000	0.008	0.125	0.015
RNSC	(6)	5	0.000	0.000	0.000	0.008	**0.400**	0.016

The "data sets" column refers to networks, where (1) denotes *PPI_Biogrid_*, (2) denotes *PPI*_*Gavin*6_, (3) denotes *PPI*_*Gavin*2_, (4) denotes *PPI_Krogan_*, (5) denotes *PPI_Ho_*, and (6) denotes *PPI_Ito_*. The best precision and recall value for each PPI network are highlighted in bold font.

#### Examples of Predicted Clusters

In this section, we present five matched and unmatched clusters predicted by CFA.

In Figure 1(A), two MIPS complexes, marked as 1 and 2, are depicted according to the protein interactions of *PPI*_*Gavin*2_. Complex 1 is an eleven- member complex (MIPS ID. 550.1.213; *Probably transcription DNA Maintanace Chromatin Structure*) that contains a protein, *Y NL*113*W*, whose interactions with other proteins are missing from *PPI*_*Gavin*2_. Complex 2 contains 12 proteins (MIPS ID. 510.40.10; RNA *polymerase II *) and there exists a protein, *Y LR*418*C*, in this complex whose interactions with other proteins are missing in *PPI*_*Gavin*2_. There are four common proteins in these two complexes. Without considering localization annotations, CFA predicts all vertices of this graph (except for *Y LR*418*C *and *Y NL*113*W*) as a 2-connected subgraph. After segregating the network using GO terms, CFA predicts two clusters (Figure 1(B)) which are matched to the real complexes in Figure 1(A).

**Figure 5:**
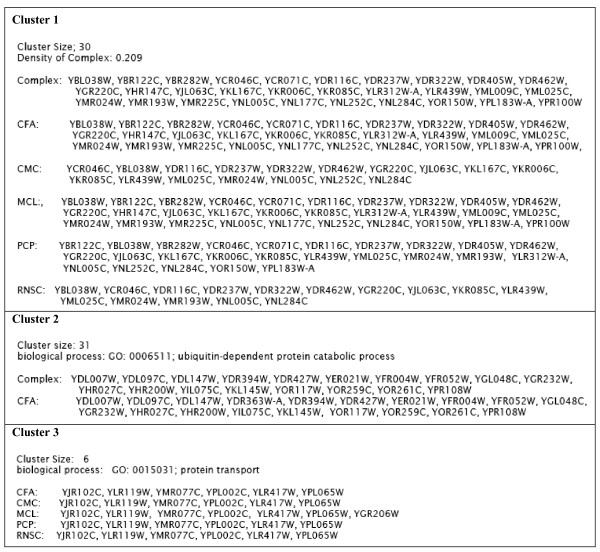
**Examples of matched and unmatched clusters**. Examples of matched (cluster 1 and 2) predicted clusters by CFA with different density. And an example of unmatched cluster predicted by CFA which contains proteins having the same specific GO annotation (GO: 0015031; *protein transport*).

In Figure 5, we show three matched and unmatched clusters. The first cluster contains 30 proteins from *PPI*_*Gavin*6_. The cluster is perfectly matched to a complex in MPC of size 30. The density in this complex is 0.2, so it can be considered as a non-dense real complex. The second cluster is a nineteen-member cluster from *PPI_Krogan_*. This cluster contains a known complex in MPC of size 18 proteins with specific GO annotation (GO: 0006511; *ubiquitin-dependent protein catabolic process*). The one additional protein (YDR363W-A) predicted by CFA to be in this cluster turns out to have the same biological process GO term annotation. We think that with more accurate experimental data, this 19th protein may also be a protein of this complex. The smallest cluster in our samples contains six proteins that are predicted by CFA in *PPI_BioGRID_*. The cluster members have the same specific GO annotation (GO: 0015031; *protein transport*), though this cluster is not presented as a known complex in MPC and APC.

To gain further insights into the differences among CFA's clusters and clusters predicted by other algorithms, we consider the first CFA cluster presented in Figure 5. This cluster is matched perfectly to a 30-member complex on MPC. In contrast, CMC's clusters only overlap with at most 16 members of this complex. The corresponding cluster predicted by PCP is a twenty five-member cluster, and the other members of the real complex do not belong to the PCP cluster. Similarly, merely fifteen members of the corresponding RNSC cluster overlap with the same complex. Among these methods only MCL predicts a cluster which is matched to the same complex perfectly.

The third cluster shown in Figure 5 is an unmatched cluster which is obtained by CFA, CMC, PCP and RNSC algorithms. None of the proteins of this cluster belongs to any real complex in MPC and APC. However, MCL predicts a cluster containing all members of the above mentioned cluster with an extra protein with a different GO term annotation.

## Conclusions

In the first part of this work, we study the impact of using informative cellular component GO term annotations on the performance of several different protein complex prediction algorithms. We have shown (Table 3) that existing algorithms predict protein complexes with significantly higher precision and recall when the input PPI network is cleansed using informative cellular component GO term annotations. Therefore, we propose for protein complex prediction algorithms a preprocessing step where the input PPI network is segregated by informative cellular component GO terms.

In the second part of this work, we study the density of protein interactions within protein complexes. We have shown (Figure 3) that there are many real complexes with different density. So density is not a good criterion for prediction of protein complexes. Therefore, we look at the connectivity number of complexes as a possible alternative criterion. We observe (Table 4) that 87%-99% of real protein complexes are 1-connected, 68%-87% are 2-connected, 35%-54% are 3-connected, and 23%-37% are 4-connected.

So in the third part of this work, we propose the CFA algorithm to predict protein complexes based on finding *k*-connected subgraphs on an input PPI network that has been seggregated according to informative cellular component GO term annotations on its proteins. Table 8 shows the precision and recall of maximal k-connected subgraphs on different PPI networks using MPC complexes as reference protein complexes. It can be seen that, by increasing the connectivity number of subgraphs, precision values show significant improvement compared to subgraphs with low connectivity numbers. However, the recall values decrease, due to a decrease in the number of predicted subgraphs. We have found that combining the *k*-connected subgraphs for various values of *k *as our set of predicted protein complexes yields the best precision vs recall performance. This combined set constitutes the predicted clusters output by CFA.

**Table 8:** Precision and recall values of maximal k-connected (*k ≥ *1) subgraphs, *C*1, *C*2, ..., *C*9, and their union *U*.

	*PPI_BioGRID_*		*PPI*_*Gavin*6_		*PPI*_*Gavin*2_		*PPI_Krogan_*		*PPI_Ho_*	
Data	Prec/Recall		Prec/Recall		Prec/Recall		Prec/Recall		Prec/Recall	
*C*1	0.356	0.163	0.486	0.248	0.685	0.252	0.537	0.184	0.423	0.112
*C*2	0.380	0.149	0.497	0.241	0.535	0.161	0.462	0.184	0.461	0.058
*C*3	0.516	0.150	0.597	0.187	0.523	0.102	0.549	0.150	0.555	0.023
*C*4	0.631	0.112	0.666	0.37	0.520	0.045	0.709	0.090	0.000	0.000
*C*5	0.615	0.094	0.666	0.070	0.538	0.022	0.720	0.065	--	--
*C*6	0.614	0.059	0.562	0.049	0.600	0.013	0.645	0.045	--	--
*C*7	0.561	0.043	0.800	0.024	0.500	0.002	0.608	0.037	--	--
*C*8	0.680	0.0353	0.714	0.018	1.000	0.002	0.666	0.033	--	--
*C*9	0.880	0.0276	0.000	0.000	--	--	--	--	--	--
*U*	0.435	0.182	0.543	0.275	0.595	0.271	0.533	0.195	0.416	0.114

**Figure 6:**
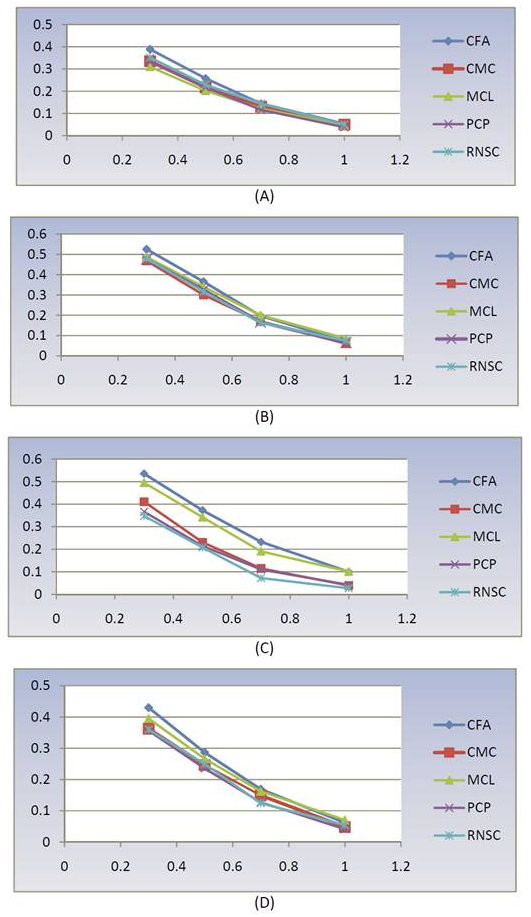
***F*-measure graphs of CFA, CMC, MCL, PCP, and RNSC**. The *F*-measure graphs of five mentioned methods by varying the threshold on matching scores for (A) *PPI_Biogrid_*, (B) *PPI*_*Gavin*6_, (C) *PPI*_*Gavin*2 _and (D) *PPI_Krogan_*.

Finally, we compare the performance of CFA to several state-of-the-art protein complex prediction methods. We have shown (Table 5) that CFA performs better than other methods for most test cases. For example, in the largest network in our test sets (*PPI_Biogrid_*), the number of complexes predicted by RNSC is very low compared to CFA. In particular, CFA predicts 19 complexes which RNSC is unable to predict, while RNSC predicts 2 complexes which CFA is unable to predict. Furthermore, by varying the threshold on the matching score, we show in Figure 6 the *F*-measure graphs based on protein clusters predicted for various protein interaction networks. We observe that CFA consistently shows the best performance compared to other methods over the entire range.

## Methods

In the Observations section we explained that cellular component annotations can help us to improve predictions. On the other hand, by studying the connectivity number of real complexes as subgraphs of PPI network, we showed that the connectivity number could be a reasonable criterion to predict complexes. So we present a new algorithm based on finding *k*-connected subgraphs (1 ≤ *k*) on PPI networks segregated by informative cellular component GO terms.

### Algorithm

A new algorithm named CFA (k-Connected Finding Algorithm) is presented here to predict complexes from an input (cleansed) PPI network. The CFA algorithm comprises two main steps. In the first step, maximal *k*-connected subgraphs for various *k *are generated as candidate complexes. In the second step, a number of filtering rules are applied to eliminate unlikely candidates.

The heart of the first step of CFA contains two simple procedures. The first procedure is *REFINE*, which removes all vertices of degree less than *k *from the input graph. This is an obvious optimization since, by the global version of Menger's theorem [34], such vertices cannot be part of any *k*-connected subgraphs. The second procedure is *COMPONENT*, which takes the refined graph and fragments it into *k*-connected subgraphs. This procedure finds a set of *h < k *vertices that disconnects the input graph, producing several connected components of the graph. The procedure is then recursively called on each of these connected components. The procedure terminates on a connected component (and returns it as a maximal *k*-connected subgraph) if it cannot be made disconnected by removing *h < k *vertices. The correctness of this procedure follows straightforwardly from the global version of Menger's theorem.

In the second step of CFA, we call the procedures defined in the first step on larger and larger values of *k *until no more *k*-connected subgraphs are returned. This way, we obtain maximal *k*-connected subgraphs for various values of *k*. These subgraphs are then filtered using the following three simple rules: (1) 1-connected subgraphs having diameter greater than 4 are removed. (2) *k*-connected subgraphs (*k *≥ 2) having diameter greater than *k *are removed. (3) Subgraphs of size less than 4 are removed. The pseudo codes of the CFA algorithm are given in Table 9.

**Table 9:** Pseudo codes of CFA

Step1:// Find maximal k-connected subgraphs

*Procedure REFINE*
Input: Graph *G *= (*V*, *E*) and a parameter *k*.
Output: All vertices in *G *of degree less than *k *are removed.
The reduced graph is returned.

*Procedure COMPONENT*
Input: Connected graph *H *= (*V*, *E*) and a parameter *k*.
Output: Fragment the graph *H *into k-connected subgraphs.
**If ***H *does not have more than *k *vertices,
**Then **stop.
Find some *u*_1,..., _*u_h _*(*h < k*) in *H *such that *H *- {*u*1,...,*u_h_*} is not a connected subgraph.
**If **such a set *u*_1_,..., *u_h _*is found,
**Then **for all connected component *c *in *H *- {*u*_1_,...,*u_h_*},
call *COMPONENT*(*c*,*k*)
**Else **return *H *as a result.

*Procedure k*-*CONNECTED*
Input: Graph *G *= (*V,E*)
Output: *COMPONENT*(*REFINE*(*G*,*k*),*k*).

**Step2:// **Filtering

*Procedure CFA*
Input: Graph *G *= (*V*, *E*)
Output: Maximal k-connected subgraphs in *G *of size at least 4.
Set *k *to 1
**While ***Ck *is not empty
Set *Ck *to the result of *k*-*CONNECTED*(G).
Increment *k*.
Set *G*1 to 1-connected subgraphs from *C*1 with the diameter *<*4.
Set *Gk *to k-connected subgraphs from *Ck *with the diameter *< k *(for *k *≥ 2)
Set *U *to the union of *Gk*'s (*k *≥ 1)
Remove all subgraphs of size less than 4 in the set *U*.

### Implementation

We choose fixed parameter values for each algorithm (Table 10). The implementations for RNSC and MCL are obtained from the main author of [42], Sylvian Brohee. The implementations for PCP and CMC are obtained from the one of their authors, Limsoon Wong.

**Table 10:** Optimal parameters for CMC, MCL, PCP and RNSC algorithms.

Algorithm	Parameter	Optimal value
MCL	Inflation	1.8
CMC	Min-deg-ratio	1
	Overlap-threshold	0.5
	Merge-threshold	0.25
	Min-size	4
PCP	FSWeight-threshold	0.4
	Min clique size	4
	Overlap-threshold	0.5
RNSC	Diversification frequency	50
	Tabu length	50
	Number of experiments	3
	Scaled stopping tolerance	15
	Shuffling diversification length	9

## Authors' contributions

LW and CE conceived the project and designed the experiments. All authors contributed to conceiving and improving the proposed algorithm. MH implemented the algorithm during all stages of its development and performed all the experiments. All authors contributed to writing the manuscript. All authors have read and approved the manuscript.
